# Function and mechanism of histone β-hydroxybutyrylation in health and disease

**DOI:** 10.3389/fimmu.2022.981285

**Published:** 2022-09-12

**Authors:** Tingting Zhou, Xi Cheng, Yanqiu He, Yumei Xie, Fangyuan Xu, Yong Xu, Wei Huang

**Affiliations:** ^1^ Department of Endocrinology, Affiliated Hospital of Southwest Medical University, Luzhou, China; ^2^ Metabolism, Metabolic Vascular Diseases Key Laboratory of Sichuan Province, Luzhou, China; ^3^ Sichuan Clinical Research Center for Nephropathy, Luzhou, China; ^4^ Cardiovascular and Metabolic Diseases Key Laboratory of Luzhou, Luzhou, China; ^5^ Department of Rehabilitation, Affiliated Hospital of Southwest Medical University, Luzhou, China

**Keywords:** β-hydroxybutyrylation, histone post-translational modifications, epigenetics, gene regulation, immune

## Abstract

Histone post-translational modifications (HPTMs) are essential epigenetic mechanisms that affect chromatin-associated nuclear processes without altering the DNA sequence. With the application of mass spectrometry-based proteomics, novel histone lysine acylation, such as propionylation, butyrylation, crotonylation, malonylation, succinylation, glutarylation, and lactoylation have been successively discovered. The emerging diversity of the lysine acylation landscape prompted us to investigate the function and mechanism of these novel HPTMs in health and disease. Recently, it has been reported that β-hydroxybutyrate (BHB), the main component of the ketone body, has various protective roles beyond alternative fuel provision during starvation. Histone lysine β-hydroxybutyrylation (Kbhb) is a novel HPTMs identified by mass spectrometry, which regulates gene transcription in response to carbohydrate restriction or elevated BHB levels *in vivo* and *vitro*. Recent studies have shown that histone Kbhb is strongly associated with the pathogenesis of metabolic cardiovascular diseases, kidney diseases, tumors, neuropsychiatric disorders, and metabolic diseases suggesting it has different functions from histone acetylation and methylation. This review focuses on the writers, erasers, sites, and underlying functions of histone Kbhb, providing a glimpse into their complex regulation mechanism.

## 1 Introduction

In eukaryotes, the core component of nucleosome is histone octamer, which is formed by two copies each of histone H2A, H2B, H3, and H4, whereas histone H1 binds to the linker DNA between nucleosomes (1). Histones amino acid residues, particularly in the tail of H3 and H4, such as lysine, arginine, serine, tyrosine, and threonine, are easily catalyzed by specific modification enzymes or enzyme complexes to form different kinds of histone post-translational modifications (HPTMs) that include methylation (Kme) (2), acetylation (Kac) (3) and phosphorylation (4). Those play an important role by regulating chromatin structure and function in determining and maintaining a specific gene expression pattern without changing the DNA sequence, ultimately affecting various biological events in eukaryotic cells. For example, histone acetylation is associated with cell cycle regulation (5), chromatin structure (6), DNA damage and repair (7), histone methylation is involved in gene repression or activation (8), and histone phosphorylation is a classical modification associated with cell division (4) and apoptosis (9).

Most frequently, HPTMs are analyzed using specific antibodies, however, despite being highly sensitive, these methods depend on the generation of specific antisera and do not allow a thorough analysis of combinations of HPTMs on the same polypeptide. Mass spectrometry (MS) is ideal for the analysis of HPTMs and is becoming the gold standard since it allows the quantification of modifications and combinations (10). Following the development of high-throughput and high-sensitivity MS, until now, more than ten types of novel HPTMs, such as lysine propionylation (Kpr) (11), lysine butylation (Kbu) (11), lysine crotonylation (Kcr) (12), lysine malonylation (Kma) (13), lysine succinylation (Ksucc) (14), lysine glutarylation (Kglu) (15), lysine 2-hydroxyisobutyrylation (Khib) (16), lysine β-hydroxybutyrylation (Kbhb) (17), lysine lactoylation (Kla) (18), lysine isonicotinylation(Kinic) (19), lysine benzoylation (Kbz) (20) and hundreds of modification sites, have been discovered in succession. Mounting evidence suggests that these novel histone marks influence gene regulation and are functionally distinct from the commonly studied HPTMs, thus adding another level of complexity to chromatin biology (21). Aberrant patterns of these novel HPTMs have been implicated in various diseases including cancer, neuropsychiatric disordered and cardiovascular disease (18, 22–24).

Ketone bodies, including acetone, acetoacetate, and β-hydroxybutyrate (BHB), are usually produced through fatty acid metabolism and utilized by extrahepatic tissues, such as heart and brain, especially during states of diminished carbohydrate availability. The failing or diabetic heart relies on ketone bodies as a source of energy (25, 26), and the use of ketogenic diets (KD) has therapeutic effects on cardiovascular homeostasis, metabolic diseases, and tumors (27). Recently, ketosis and BHB have been linked to covalent modifications, and serve as dynamic regulators of chromatin architecture and gene transcription, including acetylation and methylation, suggesting that BHB has functions beyond serving as an alternative fuel provision during carbohydrate restriction (28, 29).

In a recent landmark study, histone Kbhb marks were discovered by MS proteomics (17). BHB-induced histone Kbhb is a novel epigenetic regulatory mark that links metabolism to gene expression and provides a new avenue for studying chromatin regulation and the various functions of BHB in physiological and pathophysiological processes (30–32). Till now, histone Kbhb has been detected in yeast, flies, mice, and human cells, and a total of 46 histone Kbhb sites have been identified *in vivo* and *vitro*. Here in this review, the discovery and recent advances of histone Kbhb are described, in addition, the role and mechanism of Kbhb in health and disease are also been discussed.

## 2 The discovery and recent advances of histone Kbhb

### 2.1 The discovery of histone Kbhb

Epigenetics and HPTMs regulated by ketogenic process have long been reported in the literature (33). Evidence strongly supports that many protective effects of KD are mediated by inhibition of Class I histone deacetylases (HDACs), most likely by BHB, thereby activating expression of protective genes (34). However, in 2016, Yingming Zhao and co-workers identified that BHB works as a substrate for the histone Kbhb, which then activates gene expression, independent of acetylation. In this study, histone Kbhb marks are dramatically induced in response to elevated BHB levels in cultured cells, and in livers of mice subjected to prolonged fasting or streptozotocin (STZ)-induced diabetic ketoacidosis (17). Further confirmed by cellular experiments, the levels of histone Kbhb were elevated by BHB dose-dependent manner. The BHB itself enables Kbhb of histone lysine in the promoter regions of some starvation-related genes, helping the body to rapidly adjust and adapt to the metabolic changes (17).

**Figure 1:**
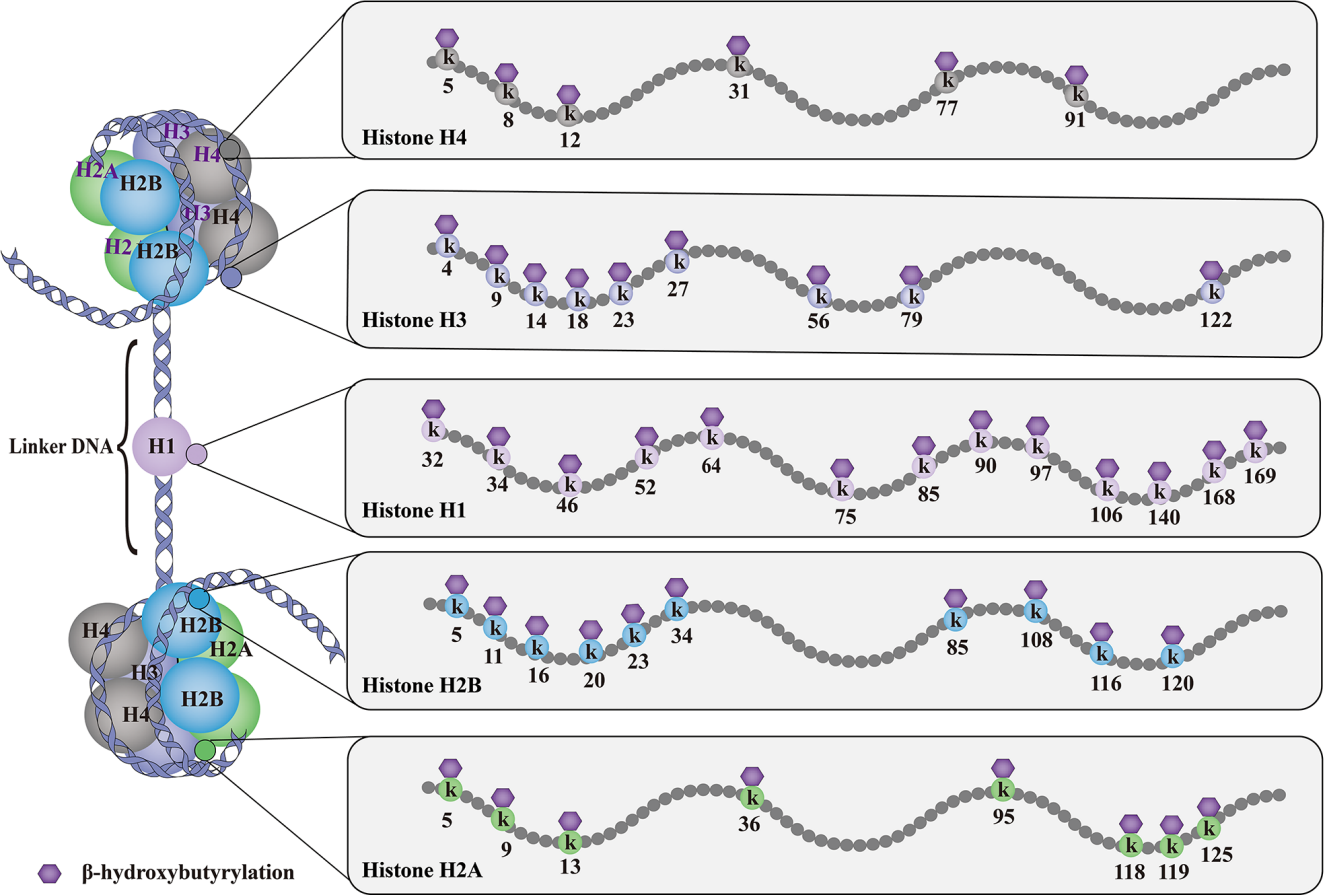
Histone Kbhb sites on histones. The basic subunit of chromatin is the nucleosome, which comprises an octamer of two copies of each of the core histone proteins (H2A, H2B, H3 and H4) wrapped by 146 bp of chromosomal DNA. Till now, histone Kbhb have been detected in four core histones (H3, H4, H2A and H2B) and linker histone H1 in yeast, flies, mice, and human cells, and a total of 46 histone Kbhb sites have been identified *in vivo* and *vitro*. These histone Kbhb modification sites cross-talk influence on other types of HPTMs dynamically in health and disease.

As shown in **Figure 1**, till now, a total of 46 histone Kbhb sites have been detected in four core histones (H3, H4, H2A, and H2B) and linker histone H1 *in vivo* and *vitro* (17, 35). 44 non-redundant histone Kbhb sites are identified in human and mouse cells (17). A total of 44 histone Kbhb sites were identified, of which 38 were from BHB-treated human HEK293 cells and 26 from livers of fasted or STZ-induced diabetic mice, indicating that Kbhb is a widespread histone mark (17).Interestingly, the histone Kbhb mark was significantly elevated in mouse liver during starvation, especially in the H3K9bhb mark localized in the gene promoter (17). Subsequently, pathways associated with the disease were extracted from the database for separate analysis and found to be involved in amino acid catabolism, circadian rhythms, redox balance, PPAR signaling pathways, and oxidative phosphorylation, as marked by elevated H3K9bhb.

Evidence from previous MS data suggests that histone Kbhb has been described not only in histone proteins but also in non-histone proteins, particularly in transcription factors and as a key enzyme in metabolism (30, 36). p53 Kbhb attenuates p53 acetylation levels, as well as the transcriptional activity of p53 at canonical p53 target genes by which ketone bodies have oncogenic roles (37). Moreover, Kbhb inhibited the activity of S-adenosyl-L-homocysteine hydrolase (AHCY) and altered the metabolite levels (30). However, this review focuses on the histone Kbhb, non-histone Kbhb are not the scope of this review.

### 2.2 The process of histone Kbhb

**Figure 2:**
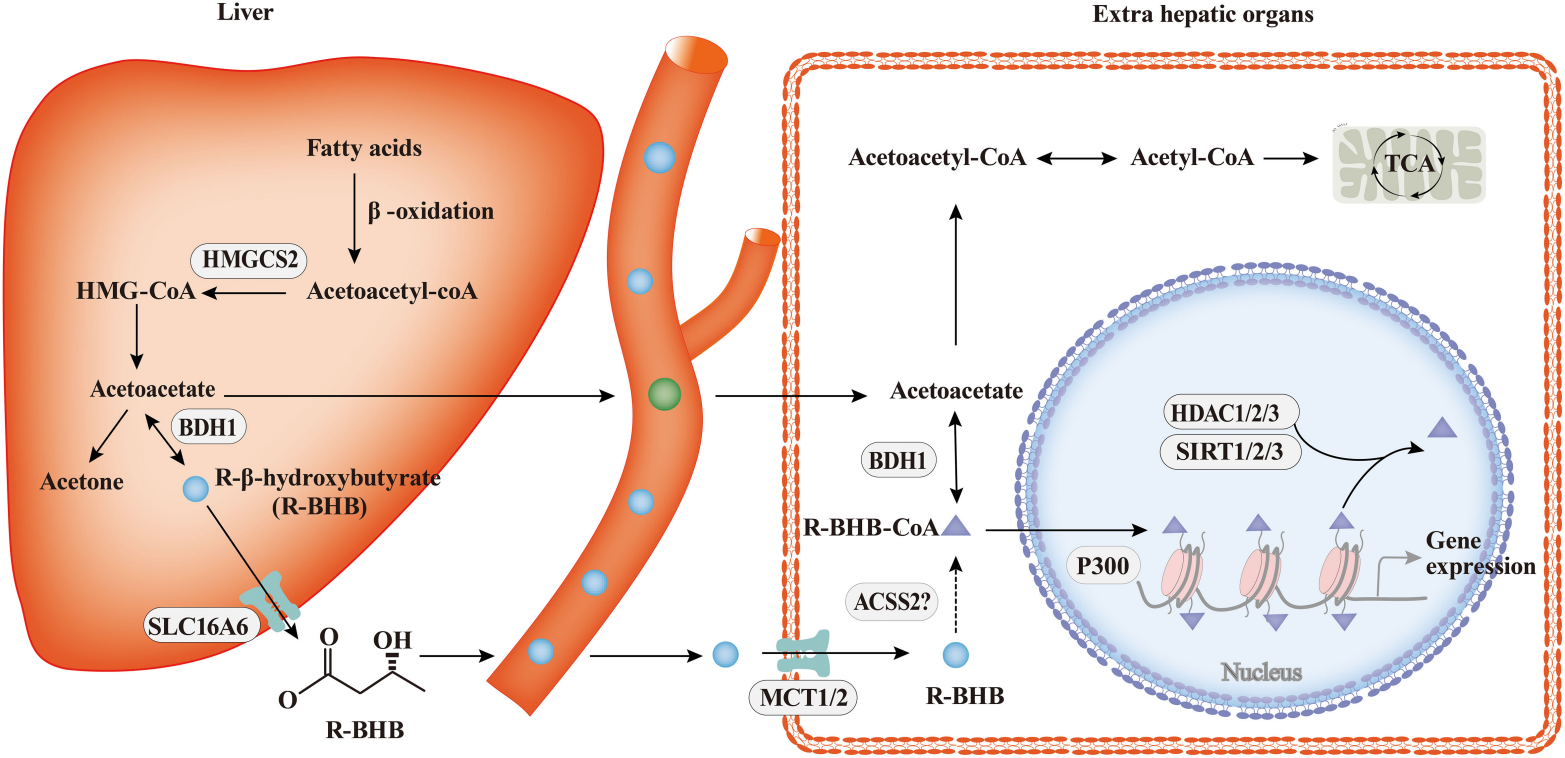
The process of ketone body metabolism and histone Kbhb. In hepatocytes of the liver, fatty acid β-oxidation generates BHB by HMGCS2 and BDH1, the key enzymes of ketone body metabolism, BHB is transported to extrahepatic organs *via* MCT1/2 and act as important sources of energy for the heart and brain during starvation by the tricarboxylic acid cycle (TCA). In addition to serving as an energy source, BHB can be converted into BHB-CoA by ACSS2, this process promotes histone Kbhb which regulate genes and mediate classical responses to carbohydrate restriction effectively. Histone Kbhb is dynamically regulated by the opposing enzymatic activities of histone acetyltransferases (HATs) and histone deacetylases (HDACs). The acyltransferase p300 act as “writer” to catalyze histone Kbhb modification, and HDAC1, HDAC2, HDAC3 and SIRT1, SIRT2, SIRT3 were reported to be the “erasers” enzymes that have been identified for de-β-hydroxybutyrylation. Histone Kbhb exerts diverse functions as described in detail below.

The process of ketone body metabolism and histone Kbhb has been shown in **Figure 2**. Fasting, exercise, calorie restriction, ketogenic diets, and any other state that induces endogenous BHB production will only produce R-type BHB(R-BHB) (38). However, S-type BHB (S-BHB) itself is not a normal product of human metabolism (39). BHB in driving histone Kbhb is provided by fatty acid β-oxidation during ketogenesis (38). Previous studies believe that BHB are transported in the blood to tissues where they are converted to acetyl-CoA, a substrate in the first step of the TCA cycle. However, the isotopic metabolic labelling shows that BHB also binds to free CoA and then form β-hydroxybutyryl-CoA (BHB-CoA), a high energy donor for histone Kbhb (40). It is worth noting that there are two potential sources of this chiral PTM, R-β-hydroxybutyrate (R-BHB) and S-β-hydroxybutyl CoA (S-BHB-CoA) (41). However, S-BHB-CoA is a transient intermediate in the last round of β-oxidation of fatty acids (42). According to the current research, we speculate that the acyl-CoA synthase short-chain family member 2 (ACSS2) is the key enzyme regulating this process because many acyl-CoAs are derived from their respective short-chain fatty acids (SCFAs) *via* ACSS2, for example, ACSS2 is thought to be the enzyme that generates crotonyl-CoA from crotonate and butyryl-CoA from butyrate (43, 44). Exhaustion of ACSS2 resulted in reducing levels of histone Kcr, demonstrating that SCFAs such as crotonate may be an endogenous source of non-acylated CoAs (45). Whether ACSS2 is responsible for the conversion of BHB to BHB-CoA has not been confirmed. Thus, the identification of ACSS2 is valuable to further elucidate the chromatin signaling pathways in which they are involved.

Chromatin modifications sensitize the genome to cellular metabolism changes, thereby establishing different functional adaptive states. The epigenome acts as a metabolic sensor, allowing rapid transcriptional adaptation to regulate cellular metabolism (46). BHB produced in fasting induces histone Kbhb, which in turn transcriptionally regulates the starvation response (17), and acetyl-CoA produced by fatty acid β-oxidation appears to be important for controlling the expression program of genes involved in lipid metabolism (47). Thus, the epigenome lies between cell metabolism and the regulation of cell fate. Notably, the enrichment and removal of these epigenetic marks are also dependent on the production of metabolites, which affect the activity of chromatin modifiers, and the enzymes in histone Kbhb pathways (40). Histone Kbhb are regulated by R-BHB in a concentration-dependent manner (17). Meanwhile, 3-Hydroxy-3Methylglutaryl-CoA Synthase 2 (HMGCS2) and β-hydroxybutyrate dehydrogenase 1 (BDH1), both of which act as key enzymes in ketone body metabolism, also affect the modification of histone Kbhb. Loss of intestinal HMGCS2 impairs the aggregation of H3K9bhb and affects H3K9bhb-related metabolic gene programs (48). However, inhibition of BDH1 resulted in the aggregation of BHB and the increase of H3K9bhb, which promoted the proliferation of hepatocellular carcinoma stem cells (49). Moreover, metabolic stress induced BHB is a natural endogenous HDAC inhibitor, which induces H3K9ac and H3K4ac (50, 51). These studies suggest that there is an interaction between cell metabolome and epigenome, which translate metabolic sensors, such as R-BHB, HMGCS2, or BDH1 into histone Kbhb and regulate metabolic reprogramming (52).

#### 2.2.1 Writers and readers

Histone Kbhb is not only influenced by the key enzymes of ketone body metabolism and metabolic substrates themselves, but also regulated by “readers”, “writers” and “erasers”. The term “writers” refers to enzymes that promote histone acylation modifications (53). Studies are currently focused on acetylation modifications and three families of histone acetyltransferases (HATS) are predominant: the p300/CREB-binding protein (p300/CBP), myst (Moz, Ybf2, SAS2, and Tip60), and GNAT (GCN5-associated N-acetyltransferase) families (54). The enzyme p300 has been identified as an acyltransferase for acetylation (55), propionylation (11) and crotonylation (56). Further experiments have shown that the Ac-CoA binding pocket of p300 can preferentially accommodate uncharged short-chain acyl-CoAs (57). According to recent studies, the acyltransferase p300 acts as a histone Kbhb “writer” to catalyze the addition of BHB to lysine, and the level of histone Kbhb at the H3K9, H3K18, H3K27, H4K8 sites decreases in response to p300 knockdown and addition of its some of these histone Kbhb sites were more sensitive to p300 knockdown than the corresponding Kac sites (35). Whether there is competition between BHB-CoA and acetyl-CoA with p300 or other mechanisms leading to greater sensitivity of histone Kbhb to p300 remains to be investigated. p300 is a co-writer of multiple acylation modifications. We speculate whether there are writers that can specifically install histone Kbhb and cause downstream alterations in different cellular events.

The “reader” proteins may specifically recognize certain HPTMs, which in turn regulate downstream gene transcription and influence cellular events. For example, three classical histone Kac readers were found to recognize croton groups bound by histone lysine residues. These include bromodomains, YEATS (Yaf9, ENL, AF9, Taf14, and Sas5), and double plant homeodomain finger (DPF) domains (58). In order to further explore the biological effects of histone Kbhb, the identification of candidate chromatin-associated proteins that are able to “readers” histone Kbhb of lysine residues is put on the agenda. However, till now, none of the unique reader proteins have been discovered for Kbhb. Given the large structural differences between histone Kbhb and other types of HPTMs, there may be reader proteins that specifically recognise histone Kbhb and cause downstream changes in different biological processes. It is probable that multiple mechanisms act synergistically in the regulation of the histone Kbhb acylation pathway.

#### 2.2.2 Erasers

Enzymes that removed acylation modifications are known as “erasers”. HDACs are classified into four classes: class I, II, and IV, HDACs are zinc-dependent, while class III HDACs (also known as sirtuins) are NAD-dependent. So far, the “erasers” of histone Kbhb are mainly the zinc-dependent HDAC1 and HDAC2 and the NAD-dependent SIRT3 (35), which mediate the removal of Kbhb through enzymatic activity. However, the activity of SIRT3 against Kbhb is dependent on the site of modification, which differs from its broad class specificity for Kcr modification (59). SIRT3 is distinguished from zinc-dependent HDACs by the fact that SIRT3 shows class-selective activity for histone de-β-hydroxybutyrylation, favoring H3 K4, K9, K18, K23, K27, and H4K16, but not H4 K5, K8, K12 (60). Moreover, lysine R-β-hydroxybutyrylation and lysine S-β-hydroxybutyrylation showed differences in their preference for the deacetylases HDAC3 and SIRT3 (41). S-BHB is stabilized by hydrogen bonding and hydrophobic contacts, but R-BHB has imperfect hydrogen bonding and weak SIRT3 binding (59). In addition, Zhao and co-workers found that HDAC3 prefers lysine R-β-hydroxybutyrylation modification compared to lysine S-β-hydroxybutyrylation *in vitro (* 61). The preference of different chiral Kbhb for deacetylases implies that we should consider the importance of the chiral structure of Kbhb. HDAC1 to HDAC3, SIRT1 and SIRT2 were recently found to have significant de-β-hydroxybutyrylation activity against core histones *in vitro* by high-performance liquid chromatography (HPLC) analysis (35). However, it is interesting that only HDAC1 and HDAC2 are histone Kbhb deacetylases in cells. In summary, HDAC1-3, and SIRT1-3 are the enzymes that have been identified for de-β-hydroxybutyrylation. However, most of the different PTMs do not occur on the same lysine residues, suggesting that a large proportion of the PTMs we studied have unique functions. Whether there are histone Kbhb specific “erasers” needs to be further explored.

## 3 The biological effects of histone Kbhb

### 3.1 Regulation of gene transcription

The involvement of histone Kbhb in the transcriptional regulation of genes affects the life activities of organisms by reprograming the epigenetic landscape on histone and modulating gene expression (See **Table 1** for details). Transcription of genes is promoted by histone Kbhb of most histones (17). To date, although a total of 46 histone sites have been identified, studies on histone Kbhb have focused mainly on H3K9bhb. For example, the mechanism of action of BHB antidepressants is related to the activation of brain derived neurotrophic factor (BDNF) by H3K9bhb (62). Histone Kbhb upregulation of vascular endothelial growth factor (VEGF) expression antagonizes the damage of aortic endothelial cells by diabetes (63). Similarly, BHB epigenetically modifies H3K9 of Foxo1, Ppargc1a (which encodes PGC-1α), and adiponectin with Kbhb, upregulating the expression of these genes (31, 65). Notably, the identified histone Kbhb sites include those lysine residues, such as H4K12, H3K4, H3K79, and H3K56, whose acetylation and methylation are essential for chromatin structure and function (23). Therefore, whether Kbhb on these histone lysine residues also affects the function of chromatin needs further investigation. In fact, chromatin is not an inert structure, and post-translational modifications of histones alter the chromatin state in response to external changes (23). In a recent study, epigenetic landscape analysis using ChromHMM and k-Means clustering revealed that fasting-induced H3K9bhb enrichment was associated with active/permissive chromatin status of H3K4me3 and H3K27ac co-enriched (48).

**Table 1:** Kbhb *in vivo* and *vitro* model of human diseases.

Study Type	Model Speciesl	Mode and Dose	Sites	Target Gene/Enzyme	Result	Ref
In *vivo*	Spatial restraint stress; Dexamethas(DEX)one-induced mouse(20mg/kg)	BHB (300mg/kg/day)by i.p; ketogenic diet	H3K9	BDNF	Antidepressant effect↑, BDNF↑,	(62)
	3-Hit strategy induced mouse and sirt3-/- mouse	ketone ester (1 mg/g/day); EMPA (10μg/g/day)	K395 of CS	CS	BDH1↓, NLRP3 inflammasome↓, BNP mRNA↓, Citrate synthase activity↑	(36)
	STZ-induced male SD rats	BHB (160, 200, and 240 mg/kg/day) by i.p	H3K9	VEGF	NO↑, VEGF↑, H3K9bhb↑	(63)
	STZ-induced male SD rats	BHB (160, 200, and 240 mg/kg/day) by i.p	H3K9	MMP-2	Serum creatinine↓, 24 h-urine protein↓, Col IV↓, MMP-2↑, H3K9bhb↑	(64)
	Not described	Fast (24,48hr); ketogenic diet (KD); 10% (w/w)3-butanedioldiet; STZ	K188, K389, K405 of AHCY	AHCY	PanKbhb↑ (liver, kidney), AHCY activity↓, AHCY-Kbhb↑	(30)
	KKAy mice (obese diabetes)	Dapagliflozin drinks freely(0.02mg/ml)	H3K9	Adiponectin	Adiponectin↑, plasma insulin↓, plasma triglyceride↓, MCP-1↓, PAI-1↓, IL-6↓, TNF-α↓	(65)
	DEX(20um)-induced Cortical neurons	BHB (10 mM) overnight	H3K9	BDNF	BDNF ↑, H3K9bhb↑	(62)
In *vitro*	Isoproterenol and NLRP3 -enriched macrophage induced H9C2 cells	BHB (5 mM)	K395 of CS	CS	COL1α1↓, COL1α2↓, COL3α1↓, CS activity↑, CS-Kbhb↑, Fatty acid uptake↓	(36)
	IL-15-induced T_mem_ cells	BHB (2, 5, 10 mM)	H3K9	Foxo1; Ppargc1a	Tcf7↑, Lef1↑ and Bcl6↑, Pck1↑, Foxo1↑, Ppargc1a↑, PGC-1α↑	(31)
	Not described	BDH1 K.O (knockout)	H3K9	Not described	H3K9bhb↑, JMJD6↑, GREB3↑, GTPBP4↑, NPM1↑, and TIMM23↑	(49)
	Not described	BHB (0.5, 2, 5, 10 mM)	K188 K389 K405 of AHCY	AHCY	PanKbhb↑, AHCY activity↓, AHCY-Kbhb↑	(30)
	3T3-L1Adipocytes	BHB (10mM), Dapagliflozin(10uM)	H3K9	Adiponectin	Adiponectin↑, MCP-1↓, PAI-1↓, IL-6↓	(65)

↑, upregulation; ↓, downregulation.H3K9bhb, histone H3 lysine 9 β-hydroxybutyrylation; BDNF, brain derived neurotrophic factor; EMPA, empagliflozin; CS, citrate synthase; BDH1, β-hydroxybutyrate dehydrogenase 1; NLRP3, nod-like receptor protein 3; BNP, brain natriuretic peptide; STZ, streptozotocin; VEGF, vascular endothelial growth factor; NO, nitric oxide; MMP-2, matrix metalloproteinase-2; Col IV, IV collagen; KD, ketogenic diet; AHCY, S-adenosyl-L-homocysteine hydrolase; MCP-1, monocyte chemotactic protein 1; PAI-1, plasminogen activator inhibitor-1; IL-6, interleukin-6; TNF-α, tumor necrosis factor-α; DEX, dexamethas; COL1α1, collagen type I α 1 chain; COL1α2, collagen type I α 2 chain; Col3α1,collagen type 3α1chain; CS-Kbhb, Kbhb of citrate synthase; IL-15, interleukin-15; Foxo1:forkhead homeobox type protein O1;Ppargc1α, peroxisome proliferative activated receptor-γ co-activator 1α;Tcf7,transcription factor 7;Lef1,lymphoid enhancer-binding factor 1;Bcl6,B-cell CLL/lymphoma 6; Pck1,phosphoenolpyruvate carboxykinase 1; JMJD6,jumonji domain containing 6; GTPBP4,GTP binding protein 4;TIMM23,translocase of inner mitochondrial membrane 23 homolog; NPMI, nucleophosmin 1

Chromatin accessibility is a chromatin property that measures the ability of chromatin-binding factors to bind to DNA, and is one of the important indicators to evaluate the structural homeostasis of chromatin (66). Previous studies have found that chromatin accessibility affects gene transcription (67). Recently, histone H3K9bhb kept chromatin in an activated state (48), suggesting a close association between histone Kbhb and chromatin accessibility. However, to date, no investigations have focused on the relationship between histone Kbhb and chromatin accessibility. “Assay for Transposase Accessible Chromatin sequencing” (ATAC-seq) is an efficient and easy to implement protocol to measure chromatin accessibility that has been widely used in multiple applications studying gene regulation (68). Further studies need to explore how histone Kbhb regulates chromatin accessibility by ATAC-seq.

### 3.2 Metabolic reprogramming

The metabolic reprogramming of cells enables them to obtain the necessary energy from an energy-deficient environment to maintain continued cell growth and proliferation. More recently, Terranova and co-workers discovered that H3K9bhb is enriched at the proximal promoter of a subset of key genes associated with lipolytic and ketogenic metabolic pathways in the small intestine cells (SICs) crypt during fasting (48). However, intestinal knockdown of HMGCS2 resulted in significant loss of H3K9bhb-related genes, suggesting that local production of BHB is responsible for chromatin reprogramming within the SI crypt. A recent study has revealed that metabolic reprogramming occurs in damaged proximal tubular epithelial cells (PTECs), characterized by a shift from lipolysis to keratolysis (69). In this study, SGLT2 inhibition-associated renal protection was mediated by the elevation of ketone bodies, which suppressed the mechanistic target of rapamycin complex1 (mTORC1) signaling (as assessed by phosphorylation of the S6 protein) hyperactivation, thereby alleviating DKD (69).

In addition, elevating the levels of histone Kbhb *in vivo* or intracellularly through the KD, starvation, dietary restriction, or direct administration of BHB regulates the activity of key enzymes in metabolism, such as AHCY (30) and citrate synthase (CS) (36), mediating disease physiopathological processes. This implies that, in the future, a KD could be used to increase BHB levels *in vivo* and regulate Kbhb of key enzymes of metabolism and important genes to slow down disease progression. The biological function of histone Kbhb should not be underestimated, as its modification itself up-regulates gene expression and indirectly regulate the level of other modifications, suggesting that the level of histone Kbhb modification can be enhanced in the future through KD or BHB interventions to regulate the transcription of disease-related genes.

## 4 Histone Kbhb in disease and therapeutics

Previous studies have shown that KD, BHB treatment, dietary restriction, and fasting enhance histone Kbhb and be involved in the progression of multiple systemic diseases. Next, we will elaborate on the role of histone Kbhb in cardiovascular diseases, neuropsychiatric disorders, kidney diseases, tumors, and metabolic diseases.

### 4.1 Cardiovascular diseases

**Figure 3:**
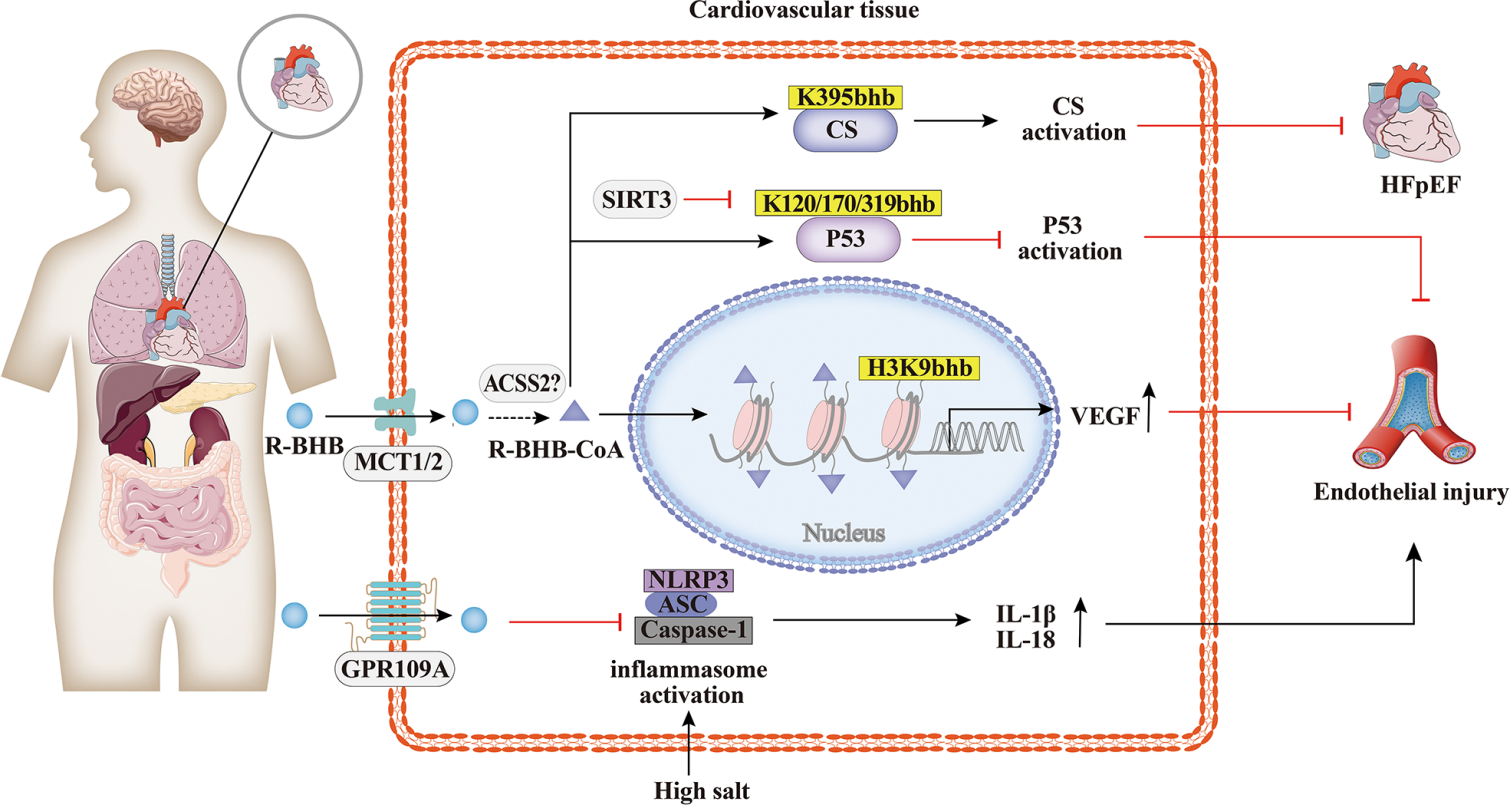
Histone Kbhb in cardiovascular diseases. Increasing studies have demonstrated the involvement of BHB and their cognate acylation reactions in cardiovascular diseases. BHB suppresses high salt induced NLRP3 inflammasome activation and then attenuates atherosclerosis and hypertension by GPR109A signaling. BHB activates citrate synthase(CS)via Kbhb modification of K395 and alleviates HFpHF. SITR3, acts as a histone de-β-hydroxybutyrylation enzyme, inhibits P53 activity thought de-Kbhb modification of K120, K170, K319 and reverses BHB-induced vascular senescence. BHB increased histone H3K9bhb, which potentially promote expression of vascular endothelial growth factor (VEGF) and antagonize aortic endothelial injury.

Cardiovascular disease is a major cause of morbidity and mortality in the world. Increasing studies have demonstrated the involvement of BHB and their cognate acylation reactions in cardiovascular diseases (70). As shown in **Figure 3**, it has been found that the histone Kbhb has affected the activation or silencing of inflammatory factor transcription. VEGF is a key factor for vascular endothelial cell integrity and function. Treatment of STZ-induced diabetic rats with different concentrations of BHB showed that BHB treatment attenuated diabetic injury of the endothelium and up-regulated the generation of VEGF. Furthermore, BHB treatment caused marked total protein Kbhb and significant elevation of H3K9bhb content in the aorta of diabetic rats. Interestingly, the ability of BHB to protect against diabetic injury of the aortic endothelium was greatest for its intermediate concentration. In summary, moderately elevated BHB could antagonize aortic endothelial injury, potentially by causing H3K9bhb to promote the generation of VEGF in diabetic rats (63). The investigators found that BHB activates CS activity *via* Kbhb modification of CS K395 (36). BHB diminished acetyl-CoA pool *via* Kbhb modification of CS and inhibition of fatty acid uptake, resulting in reduced levels of mitochondrial protein acetylation and ultimately in HFpEF mice hearts to anti-inflammatory, reduced fibrillar collagen deposition and improved mitochondrial function (36). In addition, the cardioprotective agent SIRT3 displays class-selective de-β-hydroxybutyrylation, which may be related to the cardioprotective function of SIRT3 (37). It is known that BHB administration protects from oxidative stress and inhibits inflammasome activation (50, 71). In salt-sensitive hypertension, supplementation with BHB reduces the formation of renal nod-like receptor protein 3(NLRP3) inflammasome to attenuate hypertension (72, 73). Moreover, the BHB receptor G-protein-coupled receptor 109A (GPR109A) acts a key anti-inflammatory role in various diseases, including atherosclerosis (74). Furthermore, BHB treatment suppresses oxidative stress in cardiomyocytes and prevents ischemia/reperfusion injury in the mouse heart by reducing ROS production, attenuating mitochondrial swelling, and partially restoring mitochondrial membrane potential (75). Altogether, BHB acts as a ligand to partially activate the GPR109A receptor to suppress NLRP3 inflammatory, thereby providing cardiovascular protection (74, 76). Whether the molecular mechanism of inflammatory factor inhibition by BHB is related to histone Kbhb affecting the transcriptional regulation of inflammatory factor genes need to be further explored.

### 4.2 Neuropsychiatric disorders

**Figure 4:**
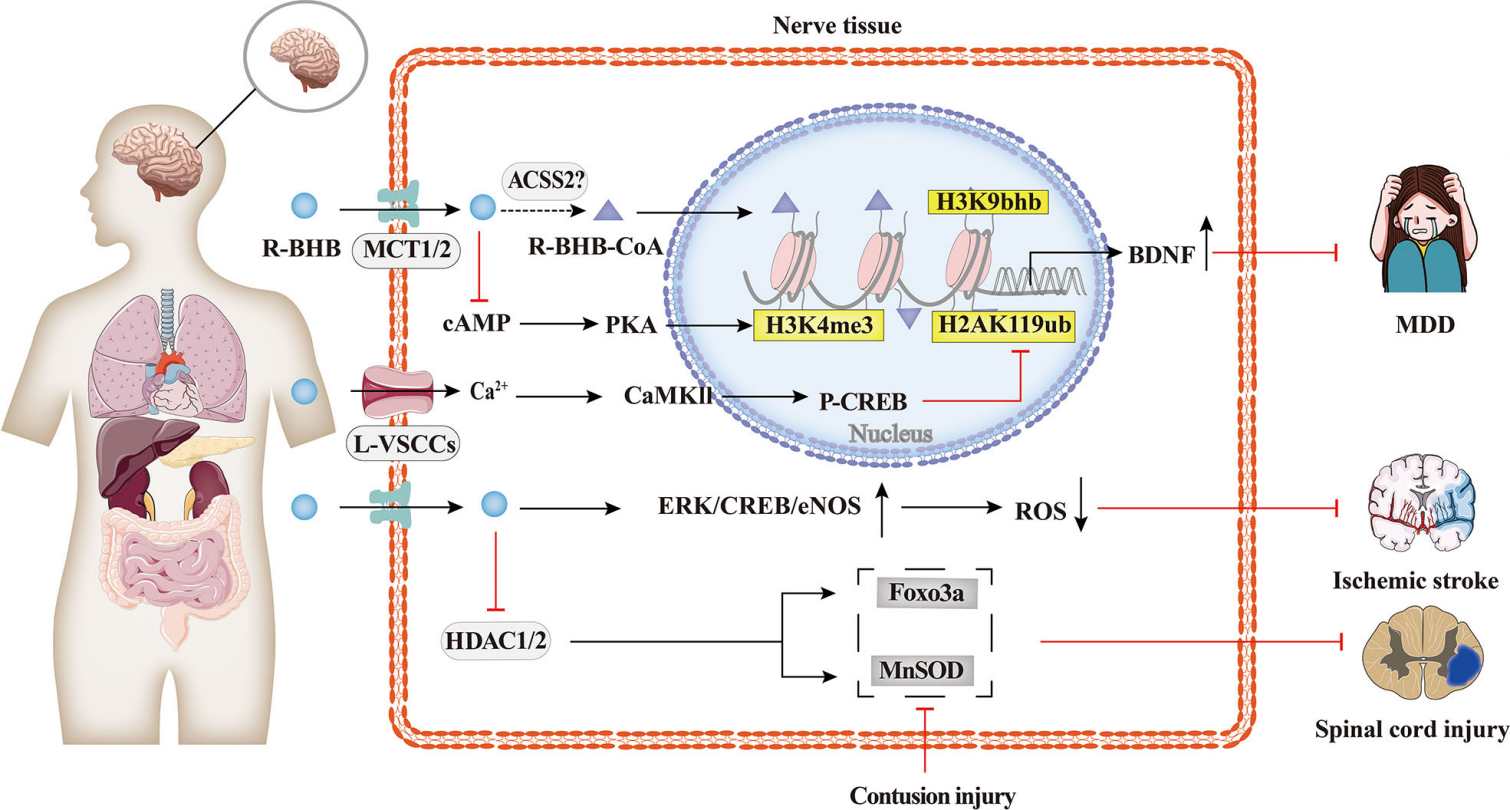
Histone Kbhb in neuropsychiatric disorders. Protective effects of BHB on the brain have been extensively researched. BHB has been found to modulate the levels of antioxidant genes, including MnSOD, Foxo3a, and glutathione, and protect neurons from contusion injury by epigenetic mechanism. BHB-reduced H2AK119ub is dependent on activation of L-type calcium channels and elevated intracellular calcium ion concentrations, which in turn activates the Ca2+/CaMKII/p-CREB signalling pathway and promotes the occupation of p-CREB and CBP in the brain derived neurotrophic factor(BDNF)promoter, thereby enhancing BDNF expression. However, BHB-increased H3K4me3 is dependent on cAMP/PKA activation. The latest research has shown that the intraperitoneal injection of BHB induced histone H3K9bhb, which promotes the expression of BDNF and alleviate major depressive disorder (MDD).

As shown in **Figure 4**, the protective effects of BHB on the brain have been extensively researched. BHB levels are elevated during KD, fasting, or vigorous exercise, and these states have been found to alleviate some of the pathological symptoms of neurodegenerative diseases such as Alzheimer’s disease, Parkinson’s disease, epilepsy, and ischemia (75, 77–79). BHB has been found to protect neurons from oxidative damage and modulate the levels of antioxidant genes, including manganese-containing superoxide dismutase (MnSOD) and forkhead box class O3a (Foxo3a), or glutathione (34, 80). Previous studies have also shown that BHB promotes BDNF expression under a normal energy supply (81). BDNF is also closely associated with psychiatric disorders, and BDNF expression is downregulated in major depressive disorder (MDD) patients, and this downregulation is rescued by antidepressants (62). Treatment of spatial restraint stress and dexamethasone-induced MDD mice with BHB showed that BHB alleviated depression and attenuated the down-regulation of BHB and H3k9bhb. The protein level of H3k9bhb but not H3k9ac was increased in a concentration-dependent way with BHB, and BHB reversed the down-regulation of BDNF *in vivo* and *in vitro*. Therefore, H3k9bhb played a crucial role in depression and was induced by BHB and then possibly activated the BDNF gene (82).

In addition, BHB enhanced BDNF expression in hippocampal neurons *in vivo* and *in vitro* by upregulating the transcriptional activation mark H3K4me3 and decreasing the transcriptional repression mark H2AK119ub. BHB-induced changes in H2AK119ub are dependent on the activation of L-type voltage-sensitive Ca2+ channels (L-VSCCs) and the elevation of intracellular, which consequently activates the Ca2+/CaMKII/p-CREB signaling pathway and promotes the occupation of p-CREB and CBP in the BDNF promoter, thereby enhancing BDNF expression. However, the increased H3K4me3 by BHB is dependent on the activation of cAMP/PKA (83).

The molecular mechanism of histone Kbhb of the BDNF promoter is one of the many potential mechanisms of histone Kbhb to protect the brain. Whether the molecular mechanism of dietary restriction to alleviate neurological disease in the KD described above is related to the modification of histone Kbhb needs to be further investigated.

### 4.3 Kidney diseases

**Figure 5:**
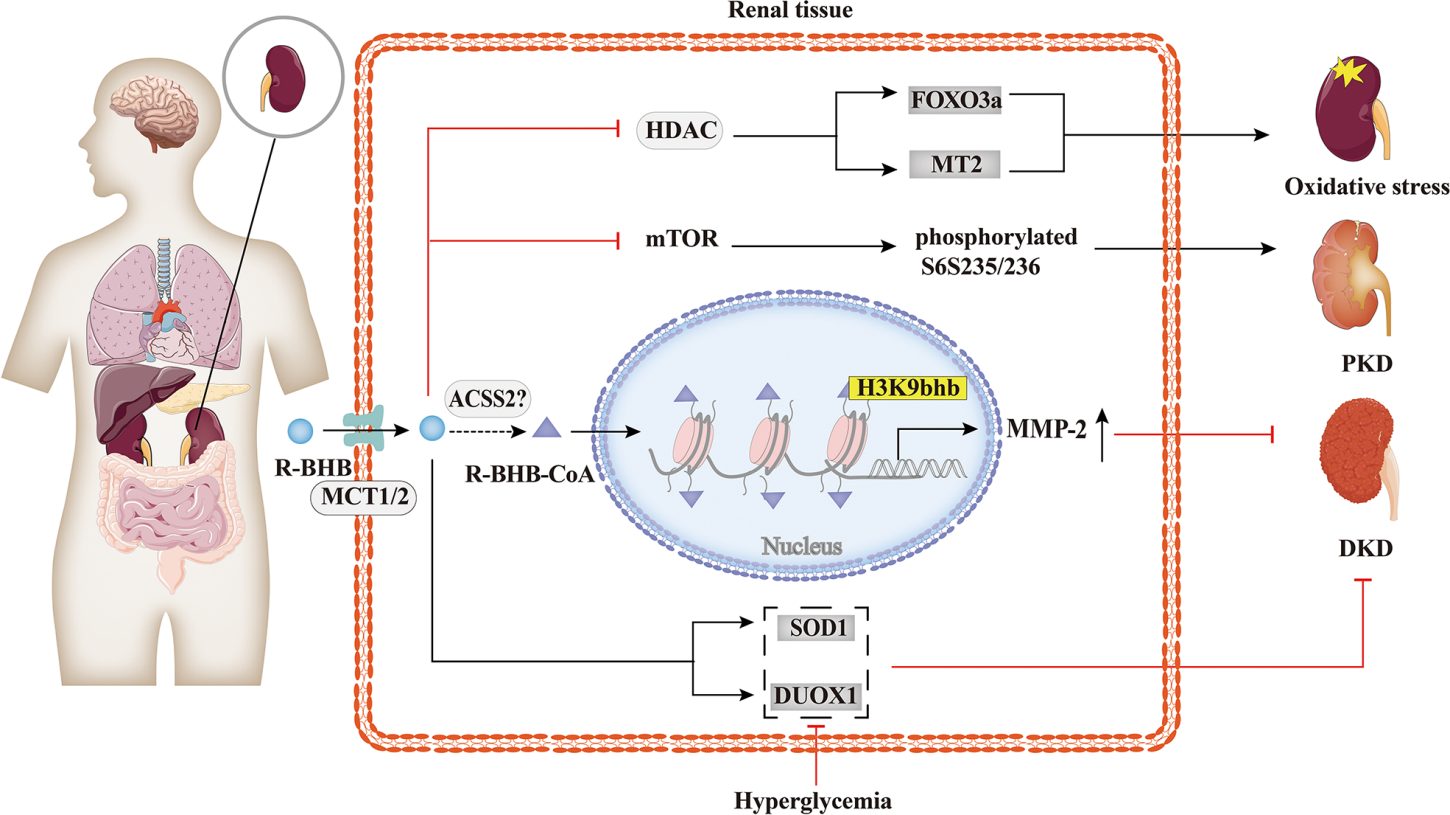
Histone Kbhb in kidney diseases. BHB has a significant protective effect against renal oxidative stress by inhibiting HDAC and enhancing FOXO3a and MT2 activity. BHB also suppressed mTOR and its downstream target, phosphorylated S6^S235/236^, markedly decreased collagen deposition and alleviated polycystic kidney disease (PKD). In addition, BHB alleviates diabetic kidney disease (DKD) by increasing the expression of SOD and DUOX1. A recent study found that BHB significantly upregulated matrix metalloproteinase-2 (MMP-2) expression by elevating H3K9bhb in the MMP-2 promoter to antagonize glomerulosclerosis in DKD rats.

While few reports have explored the relationship between histone Kbhb and kidney diseases, as summarized in **Figure 5**, evidence shows that it has a potentially beneficial effect. Increased serum BHB levels are associated with a higher probability of death in hemodialysis patients (84), but BHB inhibits oxidative stress and may be beneficial in chronic kidney disease (CKD). KD improved type 1 (Akita) and type 2 (db/db mice) diabetic kidney disease(DKD) by increasing the expression of anti-oxidative stress genes, such as Duox1 and SOD1 (85). This effect is thought to be mediated by BHB, which protects neuronal cells from glucose-induced oxidative stress, but this has not been tested in renal cells (85). Treatment of STZ-induced diabetic rats with BHB confirmed that BHB reduced serum creatinine, 24 h-urine protein, and attenuated glomerular morphological changes of the diabetic rats, with IV collagen (COL IV) content, decreased in a concentration-dependent manner. Then, BHB treatment was found to up-regulate renal matrix metalloproteinase-2 (MMP-2) generation of the diabetic rats significantly, while not affecting the increased TGF-β/Smad3 contents. Furthermore, medium and high concentrations of BHB could antagonize diabetic nephropathy injury, potentially by causing H3K9bhb to promote the generation of MMP-2 in diabetic rats (64). Moreover, it was shown that BHB inhibition of HDACs may induce oxidative stress resistance through the expression of FOXO3a and MT2 in mouse kidneys, thus improving the metabolic profile and redox status (50). In time-restricted feeding (TRF)-treated polycystic kidney disease (PKD) rats, mTORC1 activity was found to be inhibited and phosphorylation of its downstream target, phosphorylated S6^S235/236^, was significantly reduced in cystic wall epithelial cells. Therefore, BHB may slow down the progression of polycystic kidney disease (PKD) in mouse models by inhibiting the mTOR signaling pathway (86). However, further studies are needed to investigate the overall effect of histone Kbhb on CKD and the underlying mechanisms to find potential therapeutic means for CKD.

### 4.4 Tumors

**Figure 6:**
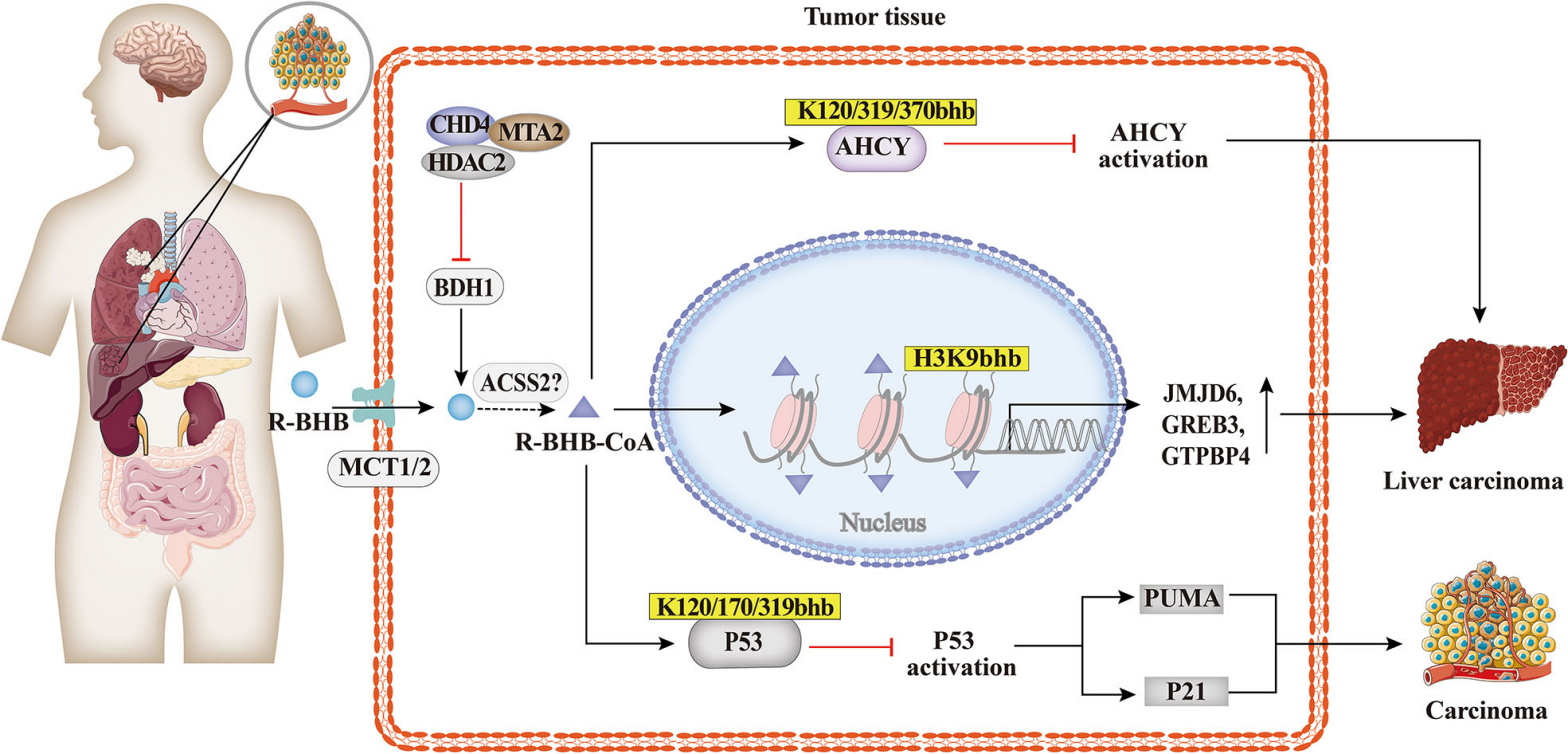
Histone Kbhb in tumors. Studies suggested that BHB or BHB induced PTMs may play an important role in the pathogenesis and treatment of tumors. MTA2-triggered R-loop trans-regulation of BDH1-mediated H3K9bhb and enhanced proliferation of hepatocellular carcinoma stem cells. BHB inhibited cyclase S-adenosyl-L-homocysteine hydrolase (AHCY) activity and promoted hepatocellular carcinoma proliferation *via* AHCY Kbhb modification of K120, K319 and K370; And then BHB inhibited P53 activity through Kbhb modification of K120, K170 and K319, which reduced the expression of the p53 downstream genes p21 and PUMA, and promoted carcinoma progression.

Carcinoma cells require large amounts of energy to support their enhanced proliferation. In contrast to non-tumor cells, tumor cells produce energy mainly through glycolysis, which is considered to be an adaptive response (87). As shown in **Figure 6**, studies suggested that BHB or BHB-induced PTMs may play an important role in the pathogenesis and treatment of tumors. The KD decreased the ratio of IGF to IGF-binding protein 3 (IGFBP3) in mouse serum (88, 89), which is associated with metabolic syndrome and cancer. The link between Kbhb of histones and hepatocellular carcinoma was also identified, and MTA2 interacts with HDAC2/CHD4 and represses BDH1 *via* R-loop transcription, leading to the accumulation of BHB and an increase in H3K9bhb, resulting in a cascade effect that promotes HCC formation and progression. H3K9bhb level was negatively correlated with BDH1. Five target genes were elevated, namely, JMJD6, GREB3, GTPBP4, NPM1, and TIMM23, which led to a poor prognosis in HCC (49). Reducing AHCY activity leads to reduced invasiveness in breast cancer and glioblastoma cell lines (90). Furthermore, non-histone Kbhb also affects the progression of tumors. Mild inactivation of AHCY activity leads to the late onset of typical disease symptoms and the pathway for the development of hepatocellular carcinoma due to AHCY dysfunction (91). While AHCY is a potential target for tumor therapy, BHB inhibits AHCY through Kbhb modification of the rate-limiting methionine cyclase AHCY K188, K389, K405, site-inhibit AHCY activity (30). The important role of P53, an important tumor suppressor whose activity is fine-tuned by post-translational modifications of P53 in tumors has been demonstrated by numerous experiments in mice in which loss of function of p53 predisposed cells to permanent damage and tumor transformation (92–94). It was recently found that BHB-mediated P53 Kbhb inhibits the level of p53 acetylation and reduces the transcriptional activity of p53 on typical p53 target genes (including p21 and PUMA), thereby reducing the effect of p53 on apoptosis and cell growth (37). The body’s energy metabolism and microenvironment are also important conditions for tumor formation and development, enlightening us about the role of histone post-translational modifications, especially histone Kbhb, lactylation, crotonylation, and other HPTMs closely related to metabolism, in tumor metabolism.

### 4.5 Metabolic diseases

**Figure 7:**
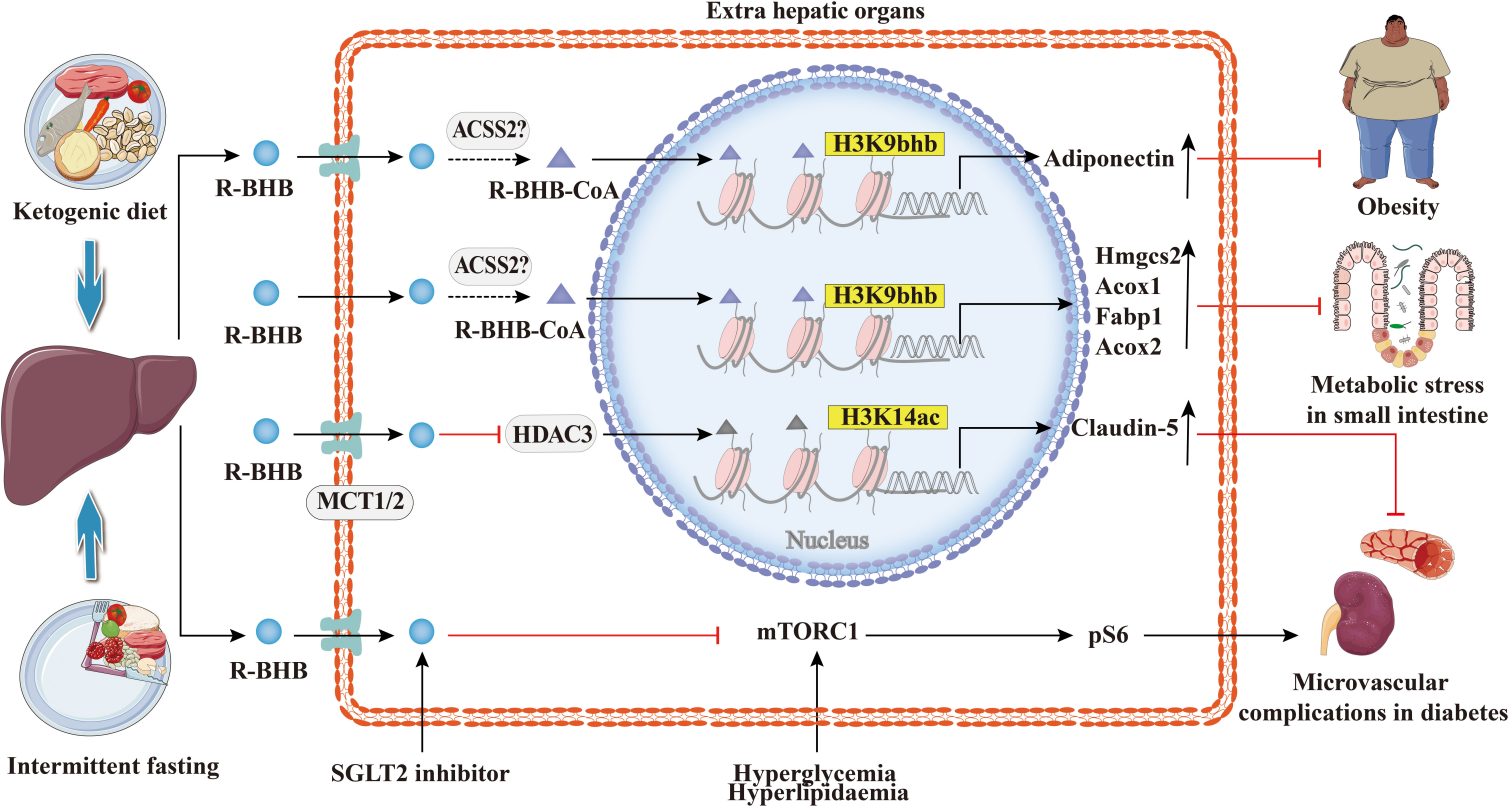
Histone Kbhb in metabolic diseases. Ketogenic diet and intermittent fasting promote BHB synthesis in the liver. BHB-induced H3K9bhb promotes adipocyte adiponectin gene expression and protects against obesity. BHB inhibits HDAC3 and leads to acetylation of H3K14 in the Claudin-5 promoter, thereby promoting Claudin-5 production and antagonizing diabetes-related cardiac microvascular hyperpermeability. Elevated BHB levels result in the enrichment of H3K9bhb within the regulatory regions of key metabolic genes in the small intestinal crypt cells. Elevated levels of BHB resulted in the concentration of H3K9bhb within the regulatory region of key metabolic genes in the epithelial cells of the small intestinal crypts. Furthermore, loss of HMGCS2 reversed the enrichment of H3K9bhb and affected the expression of H3K9bhb-related metabolic gene programs. SGLT2 inhibitors elevated BHB *in vivo*, which inhibited the mechanical target of rapamycin complex 1 (mTORC1) signaling (assessed by phosphorylation of S6 protein), resulting in alleviation of both non-proteinuric and proteinuric DKD.

The increasing prevalence of metabolic diseases such as diabetes, hyperlipidemia, obesity, and non-alcoholic fatty liver is a serious burden on human health and socioeconomics. As shown in **Figure 7**, the studies suggest that BHB or BHB-induced PTMs may play an important role in the pathogenesis and treatment of metabolic diseases and in metabolic reprogramming. Previous studies show that the treatment with SGLT2 inhibitors has been reported to be associated with high levels of serum BHB (95). In obese diabetic mice, dapagliflozin treatment resulted in elevated BHB at plasma and adipose tissue levels which mediated the promotion of apolipoprotein gene expression in adipocytes by H3K9bhb (65). Apolipoprotein has anti-inflammatory and anti-atherogenic effects (96), and BHB may be protective against obese diabetes through the H3K9bhb of the adiponectin gene. Admittedly, diabetes is a classic metabolic disease with complications that involve injury to several organs and systems, such as the kidneys and cardiovascular system (97, 98). BHB inhibits HDAC3 and leads to acetylation of H3K14 in the Claudin-5 promoter, thereby promoting Claudin-5 production and antagonizing diabetes-related cardiac microvascular hyperpermeability (28). Fasting induces an increase in H3K9bhb in small intestinal crypt cells and promotes upregulation of metabolism-related genes that may better respond to metabolic stress (48). In addition, SGLT2 inhibitors also inhibit the mechanistic target of rapamycin complex 1 (mTORC1) signaling overactivation by elevating ketone bodies *in vivo*, thereby attenuating non-proteinuric and proteinuric DKD (69).BHB inhibits adipocyte lipolysis *via* nicotinic acid receptor PUMA-G resulting in alleviation of dyslipidemia (76). Thus, histone Kbhb a novel epigenetic histone regulatory mark that links metabolism to gene expression provides a new way to investigate chromatin regulation and the different functions of BHB in important human pathophysiological states including diabetes, obesity, and hyperlipidemia.

## 5 Conclusions and prospects

Recent advances in novel HPTMs highlight a natural connection between metabolism and gene transcription. Histone Kbhb is markedly regulated by the level of BHB concentration linking starvation-responsive metabolism and epigenetic regulation. Accumulating studies identified that BHB not only acts as an alternative energy source when glucose supply is inadequate but also regulates a variety of molecular signaling functions (38). BHB and histone Kbhb may influence a wide range of human diseases such as metabolic cardiovascular disease, kidney diseases, tumors, neuropsychiatric disorders, and metabolic diseases. Histone Kbhb, as well as non-histone Kbhb widely exist and they are highly regulated by energy metabolism. Furthermore, Kbhb was recently discovered to improve the stability of the COVID-19 antibody (99). The complexity of the field of chromatin biology has been enriched with the discovery of an increasing number of novel HPTMs.

The writers and erasers play an important regulatory role in the process of histone Kbhb, acyltransferase p300 was found as a writer which catalyzes histone Kbhb, while HDAC1 to HDAC3, and SIRT1 to SIRT3 are the enzymes that remove Kbhb. These researches uncovered the key regulatory elements of the histone Kbhb pathway, laying the foundation for studying its role in different cellular processes. Until now, what are the main factors that determine the specificity and selectivity of p300, HDACs, or SIRTs enzymatic activity in the process of histone acylations are unclear. The dynamics and ratio of the intracellular concentration of BHB-CoA and acetyl-CoA may be one of the reasons, however, this hypothesis and mechanism need further research to prove. Furthermore, hyperketonemia is known to trigger ketoacidosis, but BHB or KD are used to improve metabolism or epilepsy, how to select the appropriate concentration of BHB without inducing ketoacidosis is still unknown. Therefore, prudent and well-designed clinical and basic experiments are required for a deeper and integrated understanding of histone Kbhb molecular basis in epigenetics and beyond, particularly given the relationship between and other histone acylations, and possible side effects of therapies that provoke ketosis.

## Author contributions

WH, YX conceptualized the study, collected the funds, and reviewed the manuscript. T-TZ and XC searched the literature and prepared the manuscript. YQ-H, Y-MX and FY-X were responsible for the final approval of the version to be submitted. All authors read and approved the final manuscript.

## Funding

A This work was supported by grants from the National Natural Science Foundation of China (No. 82170834, No.81970676 and No.81800741) and the Office of Science Technology and Talent Work of Luzhou (No. 2020LZXNYDP02 and No. 2021LZXNYD-G01, No. 2019LZXNYDJ40).

## Conflict of interest

The authors declare that the research was conducted in the absence of any commercial or financial relationships that could be construed as a potential conflict of interest.

## Publisher’s note

All claims expressed in this article are solely those of the authors and do not necessarily represent those of their affiliated organizations, or those of the publisher, the editors and the reviewers. Any product that may be evaluated in this article, or claim that may be made by its manufacturer, is not guaranteed or endorsed by the publisher.
